# Distributed Long-Gauge Optical Fiber Sensors Based Self-Sensing FRP Bar for Concrete Structure

**DOI:** 10.3390/s16030286

**Published:** 2016-02-25

**Authors:** Yongsheng Tang, Zhishen Wu

**Affiliations:** 1 School of Urban Rail Transportation, Soochow University, Suzhou 215137, China; tys19821025@gmail.com; 2 International Institute for Urban System Engineering, Southeast University, Nanjing 210096, China

**Keywords:** self-sensing FRP bar, Brillouin scattering-based distributed optical fiber sensing technology, long-gauge sensors, macro-strain, concrete structure

## Abstract

Brillouin scattering-based distributed optical fiber (OF) sensing technique presents advantages for concrete structure monitoring. However, the existence of spatial resolution greatly decreases strain measurement accuracy especially around cracks. Meanwhile, the brittle feature of OF also hinders its further application. In this paper, the distributed OF sensor was firstly proposed as long-gauge sensor to improve strain measurement accuracy. Then, a new type of self-sensing fiber reinforced polymer (FRP) bar was developed by embedding the packaged long-gauge OF sensors into FRP bar, followed by experimental studies on strain sensing, temperature sensing and basic mechanical properties. The results confirmed the superior strain sensing properties, namely satisfied accuracy, repeatability and linearity, as well as excellent mechanical performance. At the same time, the temperature sensing property was not influenced by the long-gauge package, making temperature compensation easy. Furthermore, the bonding performance between self-sensing FRP bar and concrete was investigated to study its influence on the sensing. Lastly, the sensing performance was further verified with static experiments of concrete beam reinforced with the proposed self-sensing FRP bar. Therefore, the self-sensing FRP bar has potential applications for long-term structural health monitoring (SHM) as embedded sensors as well as reinforcing materials for concrete structures.

## 1. Introduction

Concrete structure is the main type of infrastructures due especially to its low cost. However, concrete cracks often develop under service loads and environmental and accidental actions. In addition to decreasing structural stiffness, these cracks will speed up the corrosion of steel rebar, influencing the structure durability or safety. Therefore, damage identification, including damage detection, localization and quantification, has received considerable attention during the past two decades [1]. In order to accurately determine the damage location and severity, a suitable parameter should be chosen firstly, which must be sensitive to local damage and be a function of space coordinates. Strain field distribution can be a good indicator to interpret the local structural damage [2]. However, previous measurement incapability has prevented it from serving as an effective indicator. For large-scale concrete structures especially, the cracks present a distribution with great randomness. Thus, it is difficult to detect the cracks using the traditional “point” strain gauge.

In 1989, a new strain sensing technique was proposed based on Brillouin scattering [3], with which the strain distribution along the optical fiber (OF) can be obtained easily, capable of identifying random concrete cracks. Since then, many developments have been achieved from both lab and field applications. The optical fiber sensors based on Brillouin optical time domain reflectometer (BOTDR) were bonded on the beam surface to measure the distributed tensile strain and detect concrete cracks [4,5]. These sensors were also bonded on the steel rebar surface to monitor the inner strain [6]. Additionally, the technique was applied to get the strain field for damage identification in the destructive loading test of a pre-stressed concrete bridge [7]. Besides the beam structures, it was also used to monitor other types of structures, such as tunnel [8] and river levee [9]. The most popular BOTDR system is AQ8603 made by Company ANDO, with a maximum sensing distance of 80 km and a best strain measurement accuracy of 30 με. Meanwhile, the spatial resolution, namely the least distance for satisfying measurement needs, is 1 m, which means that the obtained strain does not reflect the structural information for some special point but for some zone about 1 m long. For detecting cracks at early age, the performance of measurement system should be improved, such as strain measurement accuracy and spatial resolution. Therefore, the techniques based on Brillouin optical time domain analysis (BOTDA) were developed due to higher strain measurement accuracy and spatial resolution [10,11]. For most BOTDA systems, the spatial resolution can be up to 0.1 m, while the strain measurement accuracy can be better than 10 με.

The BOTDA shows great advantages in structural health monitoring (SHM), especially for large-scale concrete structures. However, the strain sensing accuracy around concrete crack will still be greatly influenced by the spatial resolution even it is only 0.1 m, as the complicated strain distribution within the spatial resolution will decrease the strain measurement accuracy greatly [12]. Another problem should also be solved when the optical sensors are applied with BOTDA. It is well known that the OF is so fragile that it may be easily broken during the installation. Hence, the OF sensors often need special packaging, making the installation very complicated. The sensor durability is one more key factor to be considered when long-term SHM is conducted by bonding the OF sensors on the structure surface or steel rebar surface. Therefore, some improvement should be implemented upon the OF sensor.

In this paper, some methods were proposed to improve the comprehensive performance of the distributed optical fiber sensors. The OF sensor was designed as long-gauge OF sensor at first. Within the sensing gauge, the strain distributes uniformly along the OF to ensure satisfied strain measurement accuracy even around cracks. Then the proposed improved OF sensor was advised to be embedded into fiber reinforced polymer (FRP) bar to make a new type of self-sensing FRP bar due to some obvious advantages [13,14,15]. First, FRP will provide OF sensors with reliable and durable protection due to the excellent mechanical and chemical properties. Second, the OF sensors will make FRP self-monitoring avoid some unsatisfied failure. Lastly, it is easy to install the self-sensing FRP bar just as steel rebar. The work in references [13,14,15] has actually solved some problems, such as providing a good sensor package method with FRP. However, the OF is just bonded together with the outside FRP, named overall bonding style, which will not obviously increase the strain sensing accuracy around the concrete crack when the above distributed sensing technology is applied into concrete structure monitoring. However, in this paper, the OF sensor is not directly overall bonded with the outside FRP, but with some fixed point, named long-gauge style, which will greatly improve the strain sensing accuracy around cracks.

The research was developed around the proposed new sensor, self-sensing FRP bar, in this paper. Firstly, the measurement principle of BOTDA was illustrated as well as the key parameter, spatial resolution. Secondly, the improved distributed optical sensor was proposed using long-gauge structure, of which two key parameters, namely sensing gauge length and bonding length, were investigated and optimized. Then, the manufacturing process was introduced with two main steps. After that, the strain and temperature sensing performance of the proposed self-sensing FRP bars were verified with experiments as well as basic mechanical properties. Furthermore, the bonding performance between self-sensing FRP bar and concrete was investigated to study its influence on strain sensing. Lastly, the strain sensing performance was testified by embedding the self-sensing FRP bars into concrete beam.

## 2. BOTDA-Based Distributed OF Sensing Principle

### 2.1. Measurement Principle of BOTDA

**Figure 1:**
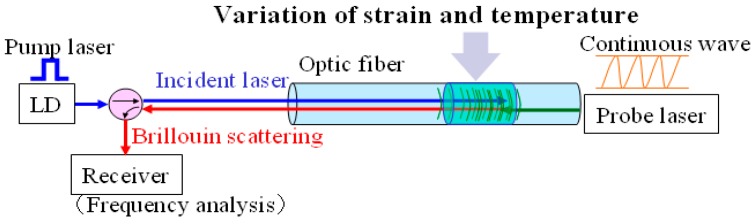
Measurement principle of BOTDA.

The BOTDA technique is based on the stimulated Brillouin back scattering, and two laser sources. One is a pulse laser (pump laser) source and the other is a continuous laser source, which are introduced into the OF from different fiber ends. When the frequency difference between the two lasers is equal to the Brillouin frequency shift, the back Brillouin scattering will be stimulated, and energy transfer will be generated between the two lasers as well. The center frequency of Brillouin back scattering will move to the location where the strain or temperature [16] change happens within the OF. Thus, the continuous strain or temperature measurement along the OF can be implemented by measuring the Brillouin frequency shift (Figure 1).

The Brillouin frequency shift *ν_В_* of OF changes in proportion to the variation of strain or temperature along the OF. The linear relationships between the Brillouin frequency shift and strain *ε* or temperature *T* are given as:

(1) $$\nu_{B}(T_{0},\varepsilon )=C_{\varepsilon }\left(\varepsilon -\varepsilon_{0}\right)+\nu_{B0}\left(T_{0},\varepsilon_{0}\right)$$

(2) $$\nu_{B}(T,\varepsilon_{0})=C_{T}\left(T-T_{0}\right)+\nu_{B0}\left(T_{0},\varepsilon_{0}\right)$$

where *C_ε_* and *C_T_* are the strain and temperature coefficients, respectively, and *T_0_* and *ε_0_* are the strain and temperature that correspond to a reference Brillouin frequency *ν_В0_*.

### 2.2. Spatial Resolution

For BOTDA, the spatial resolution is a key parameter influencing application effects of strain distribution measurement around cracks especially. Due to existence of pulse width, the received back Brillouin scattering does not reflect the information for some point of the OF but a small zone. The zone length is named as the spatial resolution, calculated as:

(3) S=ντ/2

where *ν* is the light speed in OF, and *τ* is the pulse width.

To accurately measure local strain, the spatial resolution should be improved. From Equation (3), to obtain the higher spatial resolution, the pulse with is the only parameter to be decreased. However, there will be some problems. The first is that since the phonon-field strength does not have time to reach a maximum level, the scattering amplitude decreases with increasing resolution. The weaker signal strength and consequently poorer signal-to-noise ratios (SNR) lead to lower measurement accuracy and increased acquisition times. In addition, weak signal levels limit maximum sensing lengths due to the effects of fiber attenuation. The second is that the narrow optical pulses required to obtain high resolution will have large spectral widths, far in excess of the Brillouin resonance line width. This will result in severe broadening of the measured Brillouin spectrum, causing greatly reduced accuracy in determining the Brillouin frequency. Thus, for the normal Brillouin scattering based time-domain system, the pulse width is 10 ns. In other words, the spatial resolution is not higher than 1 m.

To overcome this difficulty, a pre-pump technique has been developed, where two laser sources are introduced into OF. The main difference is in the pump laser source, which actually includes two types of pulse. One is a pre-pump laser (PL) that is applied to fully stimulate phonons, and the other is a detection pump (DP) used as detecting laser. Due to the existence of PL, the phonons can be fully stimulated before the arrival of DP. As a result, the pulse width of DP can be decreased to 1 ns without influence on the stimulation of Brillouin scattering and stimulated Brillouin gain. Accordingly, a spatial resolution of approximately cm order has been realized [17]. Based on the above technique, named as PPP-BOTDA, the Neubrex Company presents a system, NBX-6050, with a spatial resolution of 10 cm, the strain accuracy of 7.5 με and sensing distance of 2 km.

## 3. BOTDA Based Distributed Long-Gauge OF Sensor

### 3.1. Long-Gauge OF Sensor

**Figure 2:**
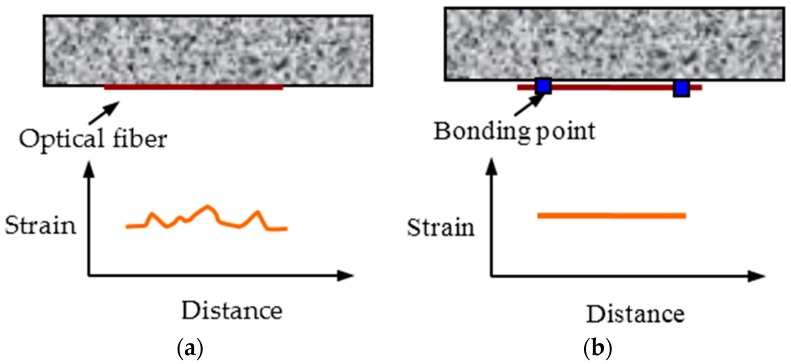
Different bonding styles for distributed optical sensor: (**a**) overall; and (**b**) fixed point.

In actual applications, there are mainly two bonding styles for distributed OF sensors (Figure 2): overall bonding and fixed point bonding. For the former, the strain along OF will be the same as the structural strain, meaning that it can reflect structural deformation accurately. However, due to the existence of spatial resolution, the strain measurement accuracy will be greatly decreased if the strain distributes complicatedly within the spatial resolution. For the latter, the strain along the OF between the two fixed points distributes uniformly, which means that it can reflect the average deformation information of the monitored zone. This long-gauge style might decrease the sensitivity a little for the small local strain change, but the basic strain measurement accuracy can be ensured due to the simple strain distribution within the spatial resolution. Thus, the distributed long-gauge OF sensor is proposed for improving the sensing performance of monitoring concrete structures especially.

### 3.2. Key Sensor Parameters

From Figure 2b, there are two key parameters for the distributed long-gauge OF sensor, namely gauge length and bonding length, to be investigated and optimized by static tension experiments. In the experiments, the BOTDA system, NBX-6050, is applied with a spatial resolution of 10 cm.

#### 3.2.1. Gauge Length

**Figure 3:**
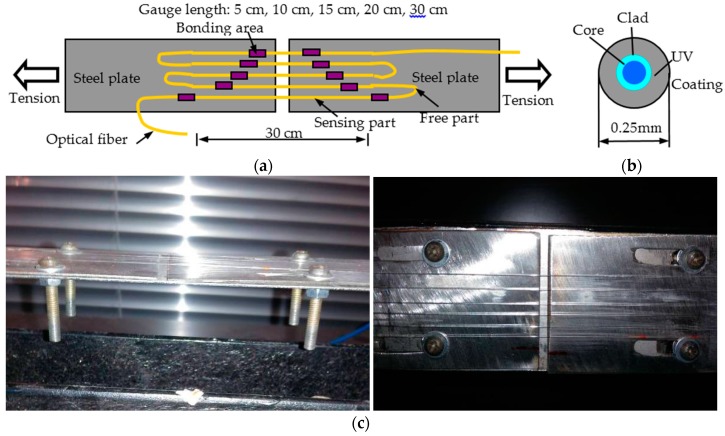
Tension experiments for distributed optical sensor with different gauge length: (**a**) sensor distribution; (**b**) OF structure; and (**c**) OF sensor and experimental setup.

In Figure 3, the OFs were bonded on the surface of two steel plates with an initial crack for simulating concrete crack. The OF applied were bare optical fiber with a diameter of 0.25 mm and its structure can be seen in Figure 3b. These OFs would be applied with different strain through moving the plates. For investigating the influence of gauge length, the length was set as 5 cm, 10 cm, 15 cm, 20 cm and 30 cm for the five sensing parts, respectively, while the bonding length was 5 cm for all the gauges, long enough to avoid slip failure. The epoxy resin was selected as the bonding material due to its excellent property.

**Figure 4:**
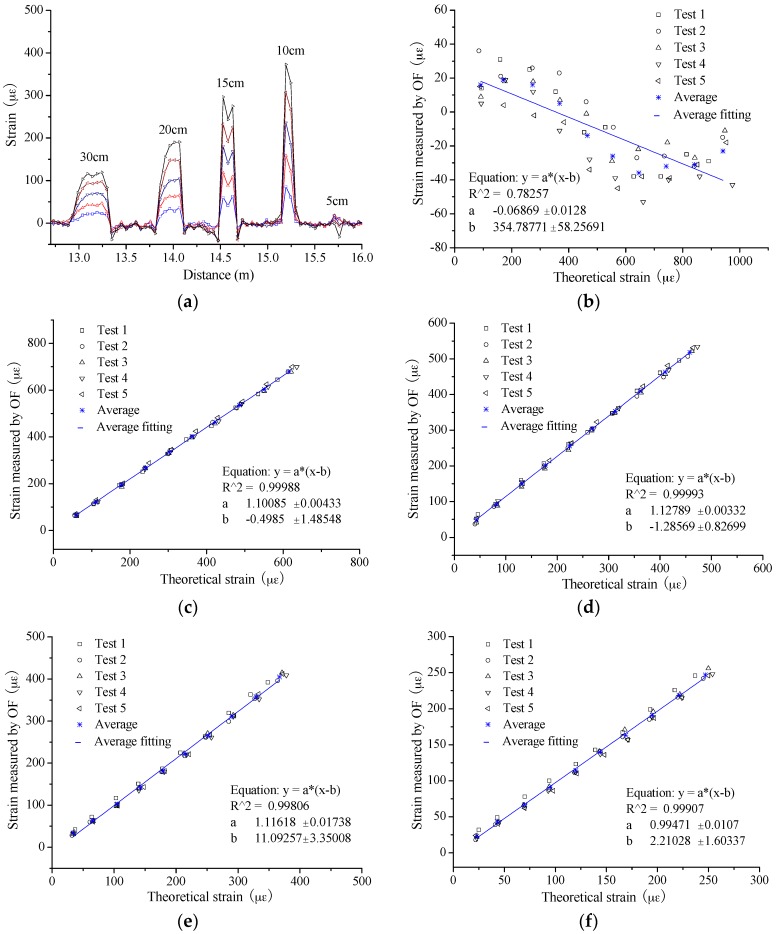
Experimental results for different gauge length: (**a**) typical strain distribution; (**b**) 5 cm; (**c**) 10 cm; (**d**) 15 cm; (**e**) 20 cm; and (**f**) 30 cm.

During the experiments, the loading and unloading were conducted five times. At the same time, the sampling distance was set as 5 cm along the OF. The typical strain distribution is as shown in Figure 4a, from which the sensing parts can be recognized from the free segments easily except the part with 5 cm gauge length. Actually, the strain value is the largest for the 5 cm gauge length; however, the BOTDA system has obviously failed to measure the strain. The only reason to explain the failure is that the sensing length is smaller than the spatial resolution and the measurement accuracy will not be promised in this condition. For the other four sensing parts, the results present some obvious trends. The larger strain change will be obtained when the gauge length is smaller. In other words, increasing gauge length will decrease strain sensitivity to some extent. However, the number of the points within the sensing part shows an inverse trend for extracting the strain average. In this paper, there are 1, 1, 2 and 4 value points applied for obtaining the average strain for each sensor of 10 cm, 15 cm, 20 cm and 30 cm, respectively. The two value points at the sides of each small “step” are excluded from the averaging process. From statistics, the higher accuracy will be obtained with larger sample number. In consideration of the two factors above, the integrated performance should be assessed.

In Figure 4b–f, the average strain from OF is compared with the theoretical value for all the sensing parts. Here, the theoretical strain is calculated by the measured crack width. In Figure 4b, the results present an obvious failure for the sensor with a 5 cm gauge length. However, from the results of the other four sensors, the excellent strain sensing properties are present, namely good linearity and repeatability. To investigate the sensing performance in detail, the slope of the fitting line is extracted between the measured strain by OF and the theoretical value and then listed in Table 1. The value of the slope will be closer to 1 when the measured strain is obtained with higher accuracy. Thus, the slope can reflect the measurement accuracy. The average values are 1.101, 1.128, 1.118 and 0.995 for these 4 sensors of gauge length 10 cm, 15 cm, 20 cm and 30 cm, respectively; in other words, the relative errors are 10.1%, 12.8%, 11.8% and 0.5%. Meanwhile, the relative standard deviation ranges from 0.6% to 1.8%, meaning a perfect sensing stability and no actual difference for all four different gauges. From the results, it is easily found that the sensor with 30 cm gauge length obtains the best integrated strain sensing performance. Thus, 30 cm is suggested as the optimized gauge length here.

#### 3.2.2. Bonding Length

**Figure 5:**
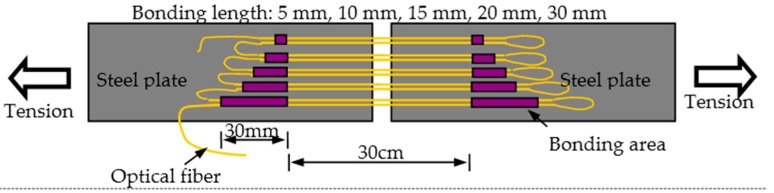
Tension experiments for distributed optical sensor with different bonding length.

**Figure 6:**
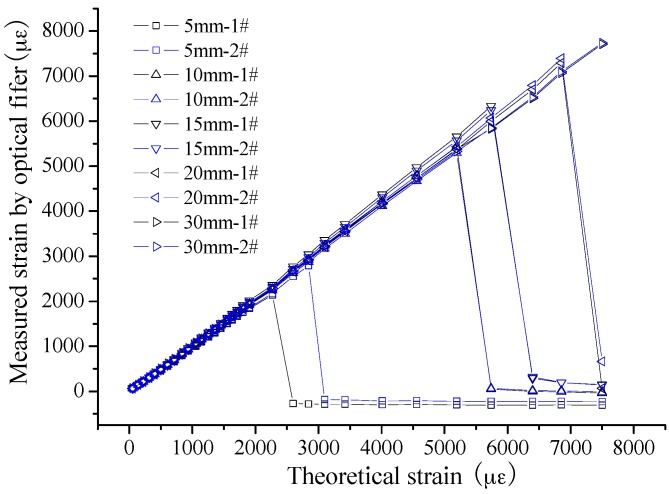
Experimental results for different bonding length.

Based on the results above, another key parameter, bonding length, is studied further. In Figure 5, 10 sensing segments in five groups were bonding on two steel plates with same sensing gauge length, 30 cm, with different bonding lengths, namely 5 mm, 10 mm, 15 mm, 20 mm and 30 mm. The monotonous tension tests were implemented to confirm the bonding performance. From the results, as shown in Figure 6, the trend can be easily found that the debonding strain is larger for the sensors with bigger bonding length. In detail, the results are as follows: 2140 µε and 2787 µε for the two specimens with 5 cm bonding length; 5434 µε and 5298 µε for 10 cm bonding length; 6333 µε and 6239 µε for 15 cm bonding length; and 7289 µε and 7395 µε for 20 cm bonding length. However, at the end of the experiments, the bond is still effective for the two specimens with 30 cm bonding length, while the strain is near 8000 µε. As is well known, the applied strain will be under 1000 µε at most during the lifetime of concrete infrastructures. Thus, the least bonding length, 5 mm, can satisfy the demands. However, in consideration of the long-term performance, such as fatigue and degradation, the bonding length is advised to not be smaller than 20 mm.

## 4. Fabrication of Self-Sensing FRP Bar with Improved Distributed OF Sensor

There are two main steps for fabricating the proposed self-sensing FRP bar, namely preparation of sensing core and manufacture of self-sensing FRP bar.

**Figure 7:**
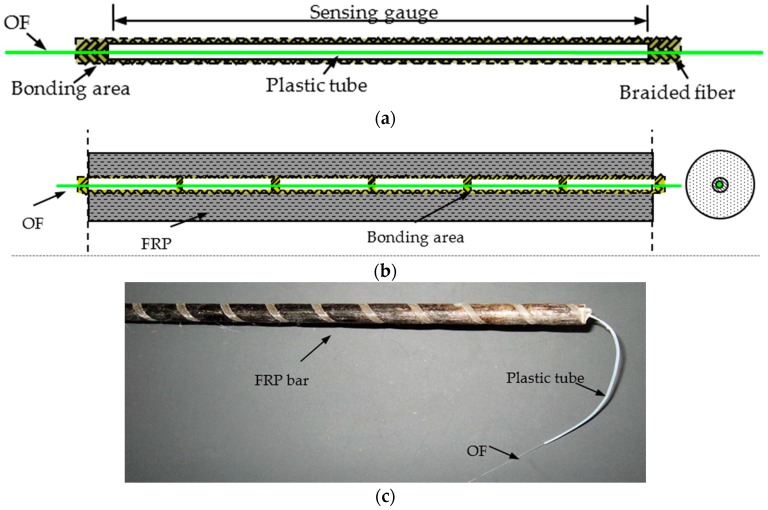
Fabrication of self-sensing FRP bar: (**a**) the unit of the long-gauge OF sensing core; (**b**) typical structure of the self-sensing FRP bar; and (**c**) the product.

In the first step, the common bare OF is first wrapped with many segments of plastic tube at, the length of which equals the sensing gauge length, as shown in Figure 7a. At the same time, the plastic tube should keep a good mechanical performance under the high temperature, 200 °C, which is the temperature of making FRP bar. This is critical for long-gauge sensors, as the strain will not distribute uniformly along OF if the tube is pressed together with the inner OF. Second, the fiber is braided around the wrapped OF. During this process, the distance between the plastic tube segments should be fixed as the same as the designed bonding length, about 2 cm–3 cm in this paper. Then, some pre-strain should be conducted upon the OF, the amount of which is determined by the actual needs. Several hundreds of micro-strains are usually enough. If the pre-strain is too large, there may be some problems with the OF sensor, such as fatigue fracture and debonding. After the pre-tension, the epoxy resin is applied to make the long-gauge sensor formed and keep the pre-strain. Then, the sensing core can be well prepared with a diameter not larger than 1 mm.

After being packaged, the sensing core is strong enough to survive the extrusion process of FRP bar manufacture. Thus, in the second step it is easy to make the self-sensing FRP bar by putting the sensing core together with the other fibers and passing the FRP bar manufacture process to be formed. However, there are still some key points to be paid attention to. First, some additional strain should be conducted upon the sensing core to overcome the contraction due to FRP bar’s cooling down. Second, the free OF should be taken from the bar to make sensor connections. Luckily, there is nearly no bond between the applied plastic tube and the outer FRP. Thus, it is easy to remove the outer FRP, as shown in Figure 7c. The typical structure and product of the bar are shown in Figure 7b,c.

## 5. Properties of Self-Sensing FRP Bar

Because of the following advantages, high ultimate strain, good environment resistance and low cost as illustrated in another paper by the author [15], the basalt fiber is applied to package the OF and manufacture the self-sensing basalt FRP (BFRP) bar. Thus, the properties studied in this paper are based on the self-sensing BFRP bar. The influence upon the properties due to different bar sizes has been already investigated in that paper [15], so the bar specimens are applied with the same diameter of 10 mm. The BOTDA system applied in the experiments is NBX-6050, with a spatial resolution of 10 cm and a strain accuracy of 7.5 με.

### 5.1. Strain Sensing Property

#### 5.1.1. Experimental Description

**Figure 8:**
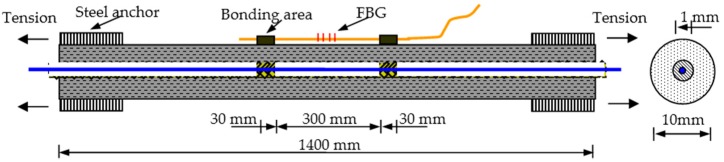
Experimental specimen and setup.

As shown in Figure 8, the self-sensing FRP bar was 1.4 m long totally, while the sensing gauge was 30 cm and located in the middle of the bar with two 30 mm long bonding areas. To implement the tensile test, two steel anchors were installed at each side of the bar with epoxy resin. The steel anchor was about 25 cm long. For comparison, one FBG (Fiber Bragg Grating) sensor was fixed on the surface of the specimen at the same position as the inner sensing gauge. Meanwhile, the gauge length and bonding length were also the same for the two types of sensors. Due to high strain sensing accuracy, the strain measurement results from FBG were considered as true values in the experiments. The FBG data were obtained by the system SM130 made by MOI Company with a strain sensing accuracy of about 1 με.

During the experiments, two loading cases were applied: Case I, small strain case; and Case II, large strain case. In Case I, the largest applied strain was about 200 µε with a loading step of about 20 µε. This case was used to simulate the structural state before concrete cracking or in the early age of cracking. In Case II, the largest applied strain was about 2000 µε with a loading step of about 200 µε. This case is used to simulate the structural state after serious damages, such as large cracks and steel rebar yielding. For each case, the test was repeated five times.

#### 5.1.2. Experimental Results and Analysis

**Figure 9:**
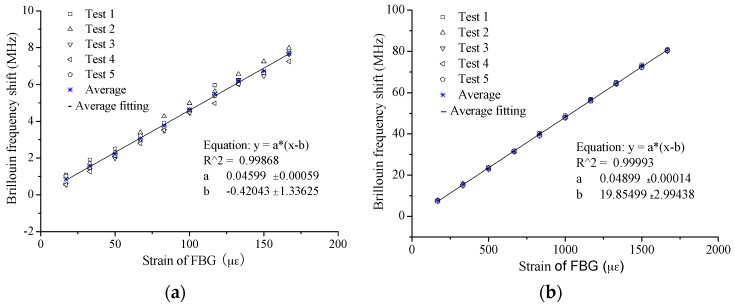
Typical experimental results of strain sensing-Specimen 2#: (**a**) small strain; and (**b**) large strain.

The typical strain-sensing results are shown in Figure 9. From the results, some distinct characteristics can be found: (1) the linear relationship between Brillouin frequency shift and strain is confirmed as all the correlation coefficients of the fitting lines are larger than 0.99; and (2) good repeatability is obviously present between the different test cycles.

For further understanding of sensing performance, the slopes, namely the strain-sensing coefficient as illustrated in Equation (1), were extracted and listed in Table 2 and Table 3 for Case I and Case II, respectively. In Case I, the average values of five tests are 45.5 MHz/0.1%, 46 MHz/0.1% and 46.9 MHz/0.1% for the three specimens. The largest relative deviation among these values is only 2.9%, which means that the strain sensing performance is stable for the different specimens. Meanwhile, the relative standard deviation of the five test cycles is not larger than 3% for each specimen, verifying a good stability and repeatability. The characteristic can also be found in Case II. The average values are 49.8 MHz/0.1%, 48.7 MHz/0.1% and 49.7 MHz/0.1% for the three specimens, among which the largest relative deviation is less than 3% and the relative standard deviation of the five test cycles is not larger than 1% for each specimen. These results have proved the excellent sensing property for large strain.

However, compared with the theoretical value, 49.7 MHz/0.1%, the coefficients obtained in Case II present a closer result. All the values obtained in Case I are smaller than the theoretical value by about 6%~8%. The same trend is found for the results of the specimens with overall bonded OF [15], as the average coefficient for specimens with a diameter of 10 mm in the small strain cases is 47.0 MHz/0.1% and 49.5 MHz/0.1% for the large strain cases. The explanation for the difference is not clear. The difference of relative strain sensing accuracy between small strain and large strain might be one main reason. Another possible reason might be the actual strain sensing properties were actually different. In actual application, the theoretical value 49.7 MHz/0.1% will be applied for convenience. Thus, it is necessary to assess the strain measurement accuracy.

**Figure 10:**
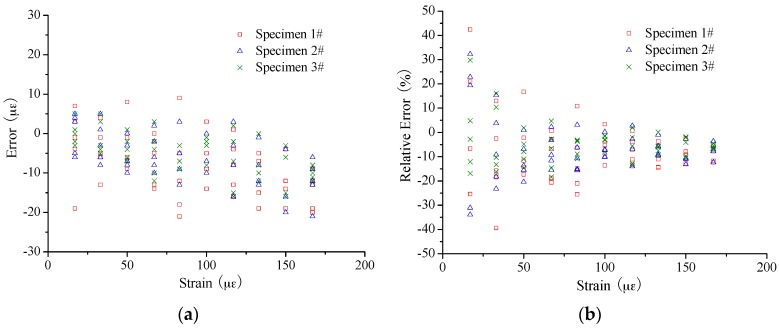
Measurement error of small strain sensing: (**a**) absolute error; and (**b**) relative error.

**Figure 11:**
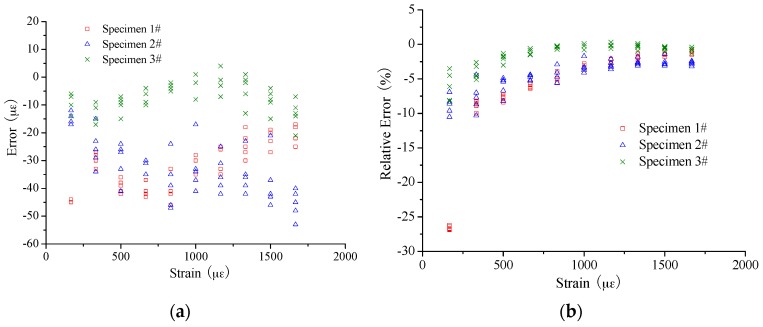
Measurement error of large strain sensing: (**a**) absolute error; and (**b**) relative error.

In Figure 10 and Figure 11, the errors were calculated to evaluate the strain sensing accuracy by applying the coefficient of 49.7 MHz/0.1%. The absolute error mainly ranges from −15 µε to 10 µε for the small strain case, showing no obvious increase with the increase of the applied strain. For the large strain case, the range of absolute error is from −50 µε to 10 µε. At the same time, the absolute error seems to present an increase with the applied strain for Specimen 2# especially. However, it is another case about the relative error. There is a trend that the relative error will decrease with the increase of applied strain. Mainly the range of relative error is as follows: −15%~10% for the strain smaller than 200 µε; −10%~0% for the strain smaller than 1000 µε but larger than 200 µε; and −5%~0% for the strain larger than 1000 µε.

### 5.2. Temperature Sensing Property

#### 5.2.1. Experimental Description

The Brillouin scattering will also be sensitive to temperature change along the OF, so the temperature sensing property should be studied for temperature compensation.

**Figure 12:**
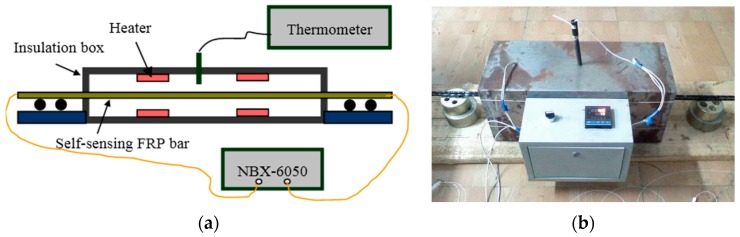
Experimental setup for temperature sensing: (**a**) sketch map; and (**b**) field picture.

The self-sensing FRP bar applied in temperature sensing tests were of the same size and OF sensor installation as that shown in Figure 8. To conduct the tests, the specimens were put into an insulation box (Figure 12), where there were four heaters to control the surrounding temperature. The temperature was measured with a thermometer, while the Brillouin frequency shifts along the specimen were measured with NBX-6050. The range of applied temperature was from 20 °C to 55 °C with a step of 5 °C. The test was repeated five times. Besides the three self-sensing FRP bars, a segment of bare free OF was also used to measure Brillouin frequency shifts for comparison.

#### 5.2.2. Experimental Results and Analysis

**Figure 13:**
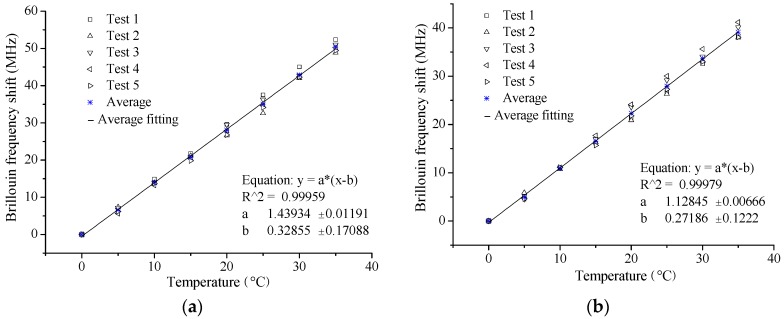
Typical results of temperature sensing: (**a**) Specimen 2#; and (**b**) Free OF.

Figure 13 shows the typical results of the temperature sensing tests. From these results, the following characteristics can be also found: perfect linear relationship between Brillouin frequency shift and temperature and good repeatability. The slope of the fitting line is the temperature sensing coefficient as illustrated in Equation (2), the results of which are listed in Table 4.

The relative standard deviation of the five tests is less than 5% for the coefficients of each specimen, proving a good temperature sensing stability of the proposed self-sensing BFRP bar. The average values are 1.488 MHz/°C, 1.439 MHz/°C and 1.537 MHz/°C for the three specimens, close to the average value, 1.403 MHz/°C, from the specimens with overall bonded OF [15]. The results have verified that the temperature sensing property is not influenced by the long-gauge package.

The Brillouin frequency shifts here are influenced by two aspects. One is the thermal expansion, and the other is that the temperature change effects the propagation of light wave. In actual application, the self-sensing FRP bar will bond together with the monitored concrete structures, meaning that the bar will present the same deformation as the outer concrete due to the thermal expansion. The strain of this part is the actual structural deformation, so it does not need to be compensated. Thus, the other part is the only part to be compensated. As the thermal expansion of OF is very small, about 0.5 µε/°C, it is reasonable to consider that the Brillouin frequency shifts obtained from the free OF are all caused by the change of light propagation. Therefore, the temperature compensation can be implemented by applying the free OF as reference.

The average temperature sensing coefficient of the free OF is 1.128 MHz/°C with a standard deviation of 0.055 MHz/°C, only 5.4% larger than the theoretical value, 1.07 MHz/°C. Considering the variance of the test itself, the theoretical value can be applied in actual monitoring application.

Furthermore, the thermal expansion coefficient can be calculated for this new type of self-sensing BFRP bar by removing the part caused by the change of light propagation. Therefore, the coefficient can be obtained as 6 µε/°C–8 µε/°C. It is close to the thermal expansion coefficient of concrete, about 10 µε/°C, so the additional stress due to temperature change will be decreased when the self-sensing BFRP bar is embedded into concrete structures.

### 5.3. Basic Mechanical Property

The basic mechanical properties were investigated as well. For elastic modulus measurement, the load was obtained with load sensors, while the strain was applied with the results from the OFs only before 2000 µε. However, the ultimate strength could be easily assessed with the load and the specimen size. In Table 5, the experimental results of elastic modulus and ultimate strength are present for comparison. From the results, the average elastic modulus is 45.6 GPa, while the average ultimate strength is 1019 MPa. The relative standard deviations are 0.3% and 3.1% for the two parameters, respectively, manifesting a good stability. Compared with the results of the similar specimens with overall bonded OF [15], the two mechanical parameters are decreased to some extent, about 1.5% and 6.9%, respectively. In other words, the mechanical performance is not remarkably influenced by the embedded sensing core. This can be well understood as the sensing core is of a diameter of about 1 mm, taking only 1% of the whole bar area.

### 5.4. Bonding Performance between Self-Sensing FRP Bar and Concrete

**Figure 14:**
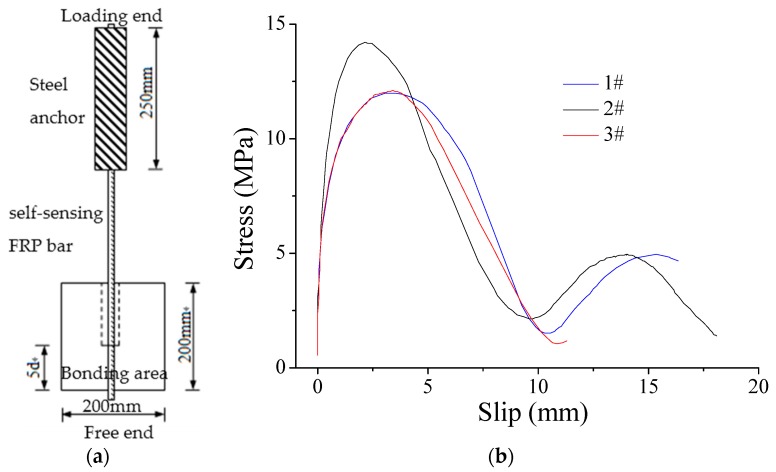
Bond test: (**a**) specimen; and (**b**) results at the free end.

The self-sensing FRP bar is designed to be embedded into concrete structures to implement the monitoring and structural reinforcing. Thus, the bonding performance between self-sensing FRP bar and concrete is considered an important parameter influencing the effects. As guided by the ACI440.3R-04, the specimens were prepared as shown in Figure 14a, with a diameter of 10 mm of self-sensing BFRP bar. The size of concrete block was 200 mm × 200 mm × 200 mm, while the bonding length was about five times the diameter of the FRP bar, namely 50 mm here. The average concrete compressive strength was 37.5 MPa. There are many parameters influencing the bonding performance, such as concrete strength, FRP bar size and FRP type. However, the simple investigation is implemented in this paper to illustrate how the bonding performance effects the sensing performance.

For the three specimens, the failure mode is the same as if the bar were pulled out from the concrete. From the results in Figure 14b, the bonding interface experiences three stages, namely no slip with load increasing, slip increasing with load increasing and load decreasing with slip increasing. For the three specimens, the trend is the same, while there are some obvious differences. The strain is 1607 με, 1383 με and 1065 με for the 1#, 2# and 3# specimen, respectively, when the slip starts. This means that the bonding interface can transfer the strain from the concrete to the self-sensing FRP bar perfectly before 1000 με. The strain at the ultimate strength is 5325 με, 6315 με and 5381 με for the three specimens, respectively. Considering the bond test results above, the bonding performance will not be a problem most time when using the proposed self-sensing FRP bar to monitor concrete structure as the strain of concrete structure is smaller than 1000 με under its service loading. However, the bonding performance should be taken into consideration, especially for some large strain cases, such as earthquakes.

## 6. Sensing Performance Verification with Concrete Beam

### 6.1. Experimental Description

**Figure 15:**
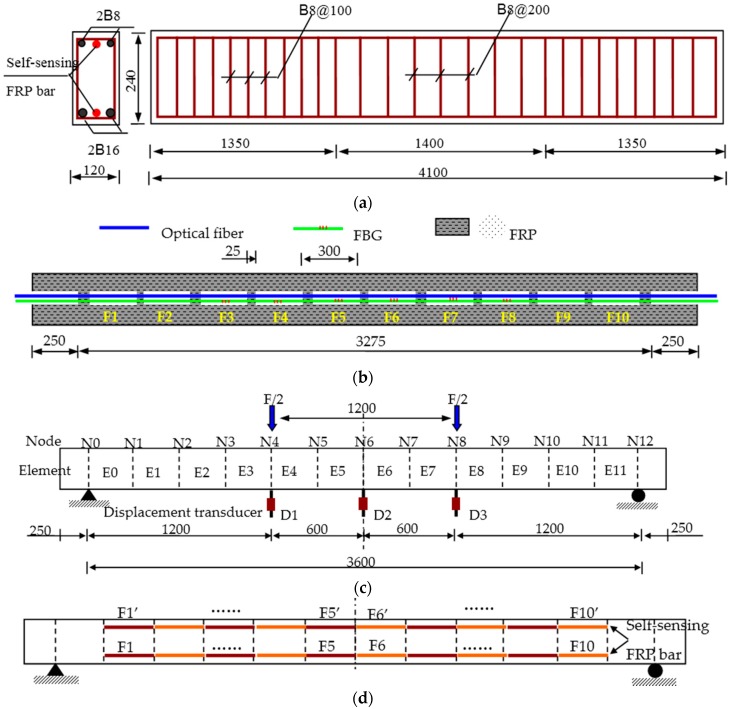
Experimental setup (Unit: mm): (**a**) beam specimen; (**b**) the self-sensing FRP bar; (**c**) loading position and displacement transducer arrangement; and (**d**) distribution of inner OF sensors along the beam.

To further verify the performance of the proposed self-sensing FRP bar, one concrete beam was prepared and reinforced with both steel rebar and self-sensing FRP bars as shown in Figure 15a. One self-sensing FRP bar was installed at the upside of beam section to measure compressive strain and the other one at the downside to measure tensile strain. As shown in Figure 15b, there were totally 10 BOTDA-based sensing gauges inside the self-sensing bar with a gauge length of 275 mm and a bonding length of 25 mm. For comparison, six long-gauge FBG sensors, namely from F3 to F8, were also embedded into the bar with same gauge parameter as the BOTDA-based sensors. In this paper, the fiber applied to make the self-sensing FRP bar is basalt fiber.

The typical loading pattern of four-points bending was applied in the static loading experiments as shown in Figure 15c. Meanwhile, three displacement transducers were installed to measure the displacement at middle span and the one-third span. Corresponding to the sensor distribution as shown in Figure 15d, the beam specimen was divided into 12 elements.

### 6.2. Experimental Results

**Figure 16:**
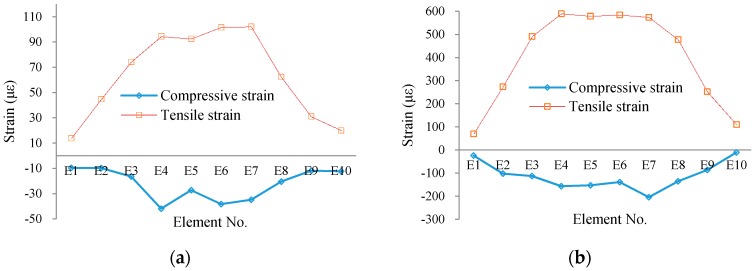
Typical macro-strain distribution at: (**a**) Load 5 kN; and (**b**) Load 10 kN.

**Figure 17:**
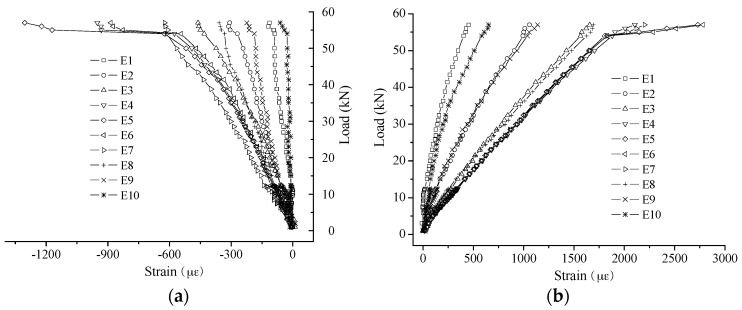
Macro-strain results: (**a**) compressive strain; and (**b**) tensile strain.

Due to the long-gauge sensing pattern, the measured strain from each sensor is macro-strain, namely the average strain of the monitored element. In Figure 16, the macro-strain results of all monitored elements are present for typical load cases before concrete cracking (Figure 16a) and after concrete cracking (Figure 16b). Moreover, all the macro-strain results are as shown in Figure 17. From the results, the key structural information can be obtained for each monitored element, such as concrete cracking and steel yielding.

**Figure 18:**
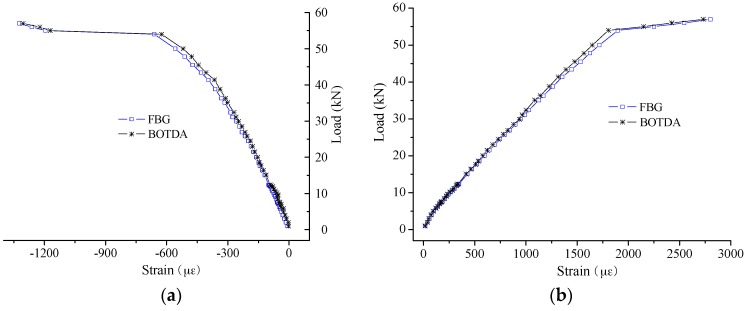
Typical strain comparison (E5): (**a**) compressive strain; and (**b**) tensile strain.

As a key indicator to assess the sensing performance of self-sensing FRP bar, the strain measurement accuracy was studied through comparing the results from FBG and BOTDA. The typical comparison results are shown in Figure 18. From the results, the high measurement accuracy is verified for both compressive strain and tensile strain measurement, as the difference between the results from FBG and BOTDA is small.

**Figure 19:**
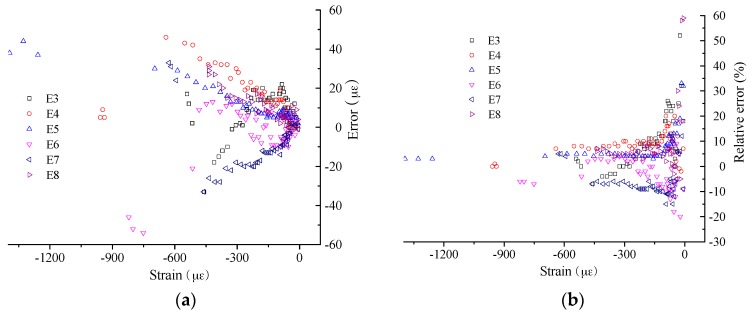
Strain measurement error for compressive strain: (**a**) absolute value; and (**b**) relative value.

**Figure 20:**
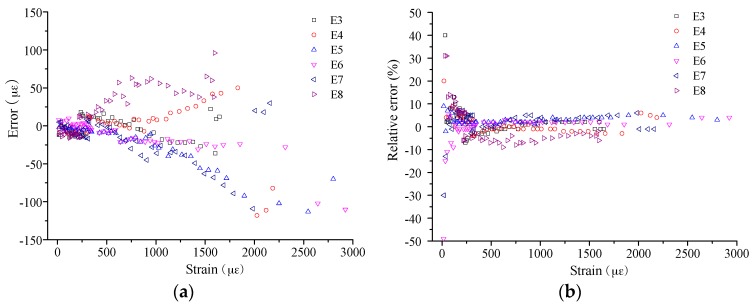
Strain measurement error for tensile strain: (**a**) absolute value; and (**b**) relative value.

In detail, the measurement errors were calculated for the BOTDA-based sensors, considering the results from the FBG as the true value. The results of measurement error are as shown in Figure 19 and Figure 20 for the compressive strain and tensile strain, respectively. For the absolute error, the trend is obvious, the error increases with increasing applied strain, while the relative error presents an opposite trend. In detail, the absolute error ranges from −60 µε to 60 µε for compressive strain measurement, and it is from −120 µε to 100 µε for tensile strain. However, the relative errors show no obvious difference between the compressive strain and tensile strain measurement, and most of them distribute from −10% to 10%. Moreover, when the applied strain is larger than 1000 µε, the relative error can be decreased to about ±5%.

**Figure 21:**
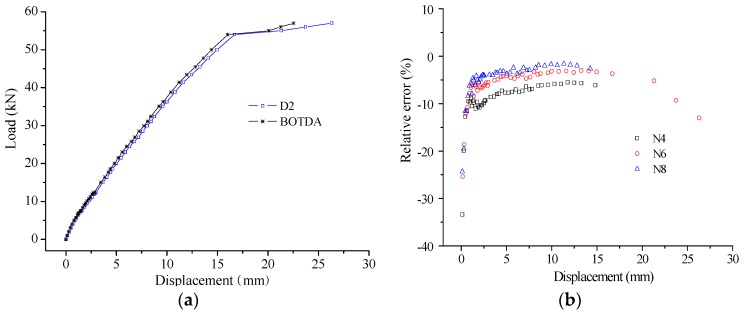
Displacement measurement: (**a**) typical results at N6; and (**b**) measurement error.

To further assess the sensing performance, another parameter is e compared. With the strain distribution, the displacement distribution can be calculated with the basic structural mechanics knowledge. Here, the strain of E0 and E11 were constructed with linear comparison with the results of E1 and E10, respectively. The typical displacement results are shown in Figure 21a. The displacement assessed by BOTDA-based self-sensing FRP bar is close to the results from displacement transducer, while there is still some difference. The relative measurement error is from −10% to 0, as shown in Figure 21b. The error is caused partially by strain sensing accuracy, and partially by the bonding performance and the analysis model especially after the steel rebar yielding. With the results here, the good sensing accuracy is verified for the proposed self-sensing FRP bar from the other side.

## 7. Conclusions and Remarks

In this paper, a new type of self-sensing FRP bar is proposed for concrete structures with BOTDA-based distributed long-gauge OF sensors to be used as both sensing and strengthening materials. For preparing the proposed self-sensing FRP bar, the distributed long-gauge OF sensor is firstly introduced as well as the industrial manufacturing process. Then, the packaged sensors are embedded into FRP to make the self-sensing FRP bar. After that, the sensing and basic mechanical properties are studied by experimentation. Moreover, the sensing performance is verified with static experiments of one concrete beam reinforced with the proposed self-sensing FRP bar. Thus, some conclusions can be drawn as follows.

(1) The BOTDA-based long-gauge OF sensor can supply reliable strain measurement accuracy, especially with the gauge length of 30 cm when the spatial resolution applied is 10 cm. Meanwhile, the bonding length is advised to not be smaller than 2 cm.

(2) The long-gauge distributed OF sensor can be well prepared with the proposed manufacturing process based on fiber package. Meanwhile, it makes the manufacture of self-sensing FRP bar much more convenient.

(3) The excellent performance of strain sensing and temperature sensing is verified with experiments, namely good linearity, repeatability and stability. The absolute strain measurement error mainly ranges from −15 µε to 10 µε for the small strain case, while it is up to −50 µε~10 µε for the large strain case. However, the relative error will decrease with the increase of applied strain. It is also proven that the temperature sensing property is not influenced by the improved sensor package. Thus, the temperature effects can be compensated using the free OF sensor.

(4) The mechanical performance of the proposed self-sensing FRP bar is not remarkably influenced by the embedded OF sensors.

(5) The bonding performance between the self-sensing FRP bar and concrete shows no influence upon the sensing performance when the strain is smaller than 1000 µε. However, it should be taken into consideration to explain the sensing accuracy when the strain is larger than 1000 µε.

(6) The advantage of sensing performance is further verified with static experiments of one concrete beam reinforced with the proposed self-sensing FRP bar. The distributed compressive strain and tensile strain can be obtained with an accuracy of about 10%. When the applied strain is larger than 1000 µε, the measurement error can be decreased to about 5%. Besides the strain distribution, the self-sensing FRP bar can monitor the displacement with an accuracy of about 10%.

However, there is still some work to be conducted in the future to make the proposed self-sensing FRP bar more useful and applicable. Firstly, the long-term strain sensing performance should be studied, such as the sensing accuracy and stability under different temperature, moisture and strain cases. Secondly, it is of great importance to ensure the long-term effective bond, especially under the fatigue loading. Thirdly, the actual performance should be investigated with filed experiments. There will be a lot of research to be implemented if the proposed self-sensing bar is used to make long-term SHM. Therefore, the work in this paper is just a good start. Due to the excellent properties, a good future can be expected for the application of the proposed self-sensing FRP bar in large-scale concrete infrastructure.

## Figures and Tables

**Table 1:** Experimental results of strain measurement accuracy.

Gauge Length	Test 1	Test 2	Test 3	Test 4	Test 5	Average	Standard Deviation
5 cm	−0.084	−0.079	−0.050	−0.076	−0.052	−0.068	0.016
10 cm	1.099	1.094	1.099	1.099	1.114	1.101	0.007
15 cm	1.117	1.119	1.142	1.115	1.150	1.128	0.016
20 cm	1.125	1.107	1.138	1.094	1.125	1.118	0.017
30 cm	1.005	0.996	1.020	0.976	0.979	0.995	0.018

**Table 2:** Experimental results of small strain sensing linearity property (MHz/0.1%).

Specimen No.	Test 1	Test 2	Test 3	Test 4	Test 5	Average	Standard Deviation
1#	45.6	45.9	46.1	46.4	44.5	45.5	0.7
2#	45.3	47.8	46.3	44.9	45	46	1.2
3#	46.3	46.1	47.4	47.1	46.7	46.9	0.5

**Table 3:** Experimental results of large strain sensing linearity property (MHz/0.1%).

Specimen No.	Test 1	Test 2	Test 3	Test 4	Test 5	Average	Standard Deviation
1#	49.6	49.9	49.7	49.8	49.9	49.8	0.1
2#	49.1	48.6	48.7	48.6	48.6	48.7	0.2
3#	49.3	49.7	49.7	49.8	49.9	49.7	0.2

**Table 4:** Experimental results of temperature sensing linearity property (MHz/°C).

Specimen No.	Test 1	Test 2	Test 3	Test 4	Test 5	Average	Standard Deviation
1#	1.541	1.468	1.513	1.439	1.479	1.488	0.040
2#	1.508	1.373	1.454	1.446	1.416	1.439	0.050
3#	1.660	1.493	1.515	1.517	1.501	1.537	0.069
Free OF	1.088	1.080	1.166	1.206	1.103	1.128	0.055

**Table 5:** Experimental results of basic mechanical property.

Specimen No.	1#	2#	3#	Average	Standard Deviation
Elastic modulus (GPa)	45.5	45.7	45.5	45.6	0.1
Ultimate strength (MPa)	1056	997	1005	1019	32
